# Resistin as Modulator of Functional Activity of Phagocytes in Colostrum and Blood of Overweight and Obese Mothers

**DOI:** 10.3390/biomedicines13112815

**Published:** 2025-11-18

**Authors:** Carla Roberta Silva Souza Antônio, Elisia Possidônea Pereira, Danielle Cristina Honorio França, Patricia Gelli Feres de Marchi, Emanuelle Carolina Honorio França, Anibal Monteiro de Magalhães Neto, Elton Brito Ribeiro, Danny Laura Gomes Fagundes-Triches, Adenilda Cristina Honorio-França, Eduardo Luzía França

**Affiliations:** 1 Institute of Biological and Health Science, Federal University of Mato Grosso, Barra do Garças 78600-000, MT, Brazildaniellechfranca@gmail.com (D.C.H.F.);; 2 Health Sciences Institute, Federal University of Mato Grosso, Sinop 78557-287, MT, Brazil

**Keywords:** obesity, oxidative stress, phagocytosis, microbicidal activity, hormone

## Abstract

**Background/Objectives**: Resistin is an adipokine involved in obesity pathogenesis, but its effects on blood and colostrum immune cells from obese mothers remain unclear. This study evaluated the functional activity of phagocytes modulated by resistin in blood and colostrum from overweight and obese mothers. **Methods**: An observational study was conducted with 82 postpartum women divided according to pregestational BMI into control, overweight, and obese groups. Blood and colostrum samples were collected to determine resistin levels and assess the functional activity of mononuclear (MN) cells. **Results**: Plasma resistin levels were higher in overweight mothers, whereas colostrum levels were lower in obese mothers. Resistin treatment enhanced superoxide release in both colostrum and blood phagocytes, independent of maternal weight status. In the presence of enteropathogenic *Escherichia coli* (EPEC), resistin-treated phagocytes from both colostrum and maternal blood showed increased superoxide production. In blood cells from overweight mothers, resistin reduced superoxide dismutase (SOD) concentration, while in colostrum, the highest SOD levels were observed in cultures of resistin-treated cells from mothers with altered weight, regardless of weight status. Blood and colostrum cells treated with resistin increased phagocytosis rates. In colostrum, resistin-treated cells from eutrophic mothers showed high microbicidal indices, whereas cells from mothers with altered weight showed reduced microbicidal indices. In colostrum cells, adipokine levels were reduced in the obesity group. **Conclusions**: Resistin modulates oxidative metabolism and the functional activity of blood and colostrum phagocytes across all maternal weight statuses, suggesting a possible role for resistin in the maternal immune response associated with obesity.

## 1. Introduction

Obesity is a major global public health challenge that contributes significantly to increased morbidity and mortality rates. It results from an imbalance between caloric intake and energy expenditure and is considered a chronic noncommunicable disease caused by interactions between genetic, environmental, neural, immunological, and hormonal factors [1,2,3].

During pregnancy, maternal obesity markedly increases the risk of obstetric and perinatal complications. Women who experience excessive gestational weight gain are more likely to develop postpartum obesity, which may, in turn, affect the health of the newborn by altering growth patterns and increasing the risk of chronic diseases later in life [4].

Adipose tissue, once regarded merely as an energy reservoir, is now recognized as a dynamic endocrine organ capable of secreting a variety of bioactive molecules, including hormones and cytokines known as adipokines. These mediators play essential roles in regulating energy metabolism, inflammatory responses, and insulin sensitivity [1,5]. In obesity, adipose tissue expansion is accompanied by increased expression and secretion of these molecules, influencing processes such as cell differentiation, hematopoiesis, and immune modulation [5].

Among the adipokines, resistin has emerged as a molecule of particular interest. Resistin is a 12.5 kDa cysteine-rich peptide belonging to the resistin-like molecule (RELM) family and is secreted by adipocytes, monocytes, macrophages, and splenic cells. Elevated levels of this adipokine have been consistently observed in individuals with obesity [6]. Beyond its systemic metabolic effects, resistin has also been detected in human milk, where it may play a regulatory role in endocrine function and energy metabolism of the newborn. Given its bioactive nature, resistin may influence body composition and growth during early life [7].

Studies report interactions between maternal obesity, soluble and cellular components of colostrum, and immune modulation. Resistin has been shown to modulate macrophage function in the colostrum of obese and diabetic mothers, restoring their microbicidal capacity and reducing apoptosis in these cells [8]. Likewise, a metabolomic and microbiome-based analysis revealed distinct co-occurrence networks of metabolites, microbiota, and cytokines in the colostrum of obese mothers, underscoring the metabolic and immunological remodeling that occurs in this context [9]. Additionally, adipokines such as leptin and adiponectin, along with melatonin, have been shown to modulate lymphocyte activity in the colostrum of obese mothers, indicating the intricate interplay between metabolic and immune pathways during lactation [10].

Maternal hypercaloric diets can alter the synthesis and regulation of key metabolic hormones during pregnancy, notably leptin, whose dysregulation has been associated with an increased risk of future cardiometabolic diseases. However, the underlying mechanisms linking maternal obesity with postnatal metabolic and immune alterations remain poorly understood [11].

The neonatal immune system, although capable of mounting immune responses, is functionally immature at birth. Breastfeeding provides essential immunological protection against environmental pathogens and helps compensate for this immaturity [12]. Colostrum, the first form of milk produced after delivery, is rich in bioactive compounds—including proteins, lipids, carbohydrates, minerals, hormones, and immune cells—that are fundamental for neonatal growth and immune system development [13,14]. The synergistic interaction among its immunomodulatory and antimicrobial components contributes to protection against infectious diseases during early childhood [15,16].

Given that obesity influences both metabolic and immune functions, and considering the intricate immunological and nutritional relationship between mother and child during pregnancy and lactation, resistin may play a key role in mediating these effects. Therefore, the present study aimed to evaluate the influence of resistin on the modulation of functional activity in colostrum and blood phagocytes from mothers with overweight and obesity.

## 2. Materials and Methods

### 2.1. Study Design and Participants

A cross-sectional study was conducted with 82 clinically healthy donors enrolled at the Municipal Hospital of Barra do Garças, MT, Brazil, in 2019. Participants were divided into three groups based on pregestational body mass index (BMI): normal BMI (18.5–24.9 kg/m^2^, *n* = 28), overweight (BMI ≥ 25 and <30 kg/m^2^, *n* = 27), and obese (BMI ≥ 30 kg/m^2^, *n* = 27).

Inclusion criteria were age between 18 and 35 years; known pregestational weight or measured by the 13th gestational week; gestational age at delivery between 37 and 41 6/7 weeks; negative serology for hepatitis, HIV, and syphilis; absence of prenatal or dietary restrictions; and signed informed consent. Exclusion criteria included gestational diabetes, twin pregnancy, fetal malformations, and preterm delivery before 36 weeks.

The study was approved by the institutional Research Ethics Committee (CAAE 45102815.3000.5587, Opinion No. 1.064.829). Written informed consent was obtained from all participants before enrollment.

### 2.2. Blood Sampling and Cell Isolation

Maternal blood (8 mL) was collected before labor in anticoagulant tubes from the participant. Samples were centrifuged at 160× *g* for 15 min to separate plasma and cells. Mononuclear cells were isolated using a Ficoll-Paque gradient (Pharmacia, Uppsala, Sweden), yielding preparations with 95% purity, verified by light microscopy. Purified monocytes were resuspended in serum-free Medium 199 at 2 × 10^6^ cells/mL and used immediately for assays of superoxide release, phagocytosis, and microbicidal activity. Plasma was stored at −80 °C for subsequent analysis of resistin.

### 2.3. Colostrum Sampling and Cell Isolation

Approximately 8 mL of colostrum was collected from each participant between 48 and 72 h postpartum in sterile plastic tubes. Samples were centrifuged at 160× *g*, 4 °C, for 10 min, separating three phases: lipid-containing supernatant, intermediate aqueous phase, and cell pellet. The lipid layer was discarded, and the aqueous phase was stored at −80 °C. Mononuclear cells were isolated from the pellet using Ficoll-Paque, achieving 98% purity, and resuspended in serum-free Medium 199 at 2 × 10^6^ cells/mL. Cells were immediately used for functional assays. The colostrum supernatant was stored at −80 °C for later resistin measurement.

### 2.4. Resistin Quantification

Resistin levels in plasma and colostrum supernatant were determined using a Human Resistin ELISA kit (Sigma, St. Louis, MO, USA; Catalog NºRAB0419). All samples were tested in duplicate, and the assay had an inter-assay CV < 12%, an intra-assay CV < 10%, and a sensitivity of 0.16 ng/mL, with a standard curve range of 0–10 ng/mL. Absorbance was measured at 450 nm, and concentrations were calculated from a standard curve, expressed in ng/mL.

### 2.5. Treatment of Colostral and Blood Phagocytes

To assess the effects of resistin on superoxide anion release, superoxide dismutase, phagocytic activity, microbicidal activity, and intracellular Ca^2+^ release, mononuclear phagocytes (2 × 10^6^ cells/mL) were incubated with 50 µL of resistin (100 ng/mL, Sigma, St. Louis, MO, USA) according to Dalcin et al. [8] for 60 min at 37 °C. Cells were washed once with Medium 199 at 4 °C and used immediately in assays. Control samples received Medium 199 only.

### 2.6. Escherichia Coli Strain

We used enteropathogenic *Escherichia coli* (EPEC) to assess the functional activity of colostrum and blood phagocytes. This bacterium was isolated from the stool of an infant with acute diarrhea (serotype 0111: H^−^ AL^−^, *eae^+^*, *eaf^+^*, *bfp^+^*). The EPEC was prepared and adjusted to 10^7^ bacteria/mL.

### 2.7. Superoxide Release Assay

Superoxide anion release was determined by the cytochrome C reduction method [17]. Mononuclear phagocytes and bacteria were incubated for 30 min to allow phagocytosis, then resuspended in PBS containing 2.6 mM CaCl_2_, 2 mM MgCl_2_, and 2 mg/mL cytochrome C. Suspensions (100 µL) were incubated at 37 °C for 60 min, and Absorbance was measured at 550 nm. Results were expressed as nmol O_2_^−^. All experiments were performed in duplicate.

### 2.8. CuZn-Superoxide Dismutase (SOD) Activity

SOD activity in colostrum supernatant was measured using the nitroblue tetrazolium (NBT) assay [18] and spectrophotometrically read at 560 nm. Plasma or standard solutions were mixed with chloroform–ethanol (1:1 *v*/*v*), NBT, and EDTA solutions. pH was adjusted to 10.2 with carbonate buffer plus hydroxylamine. Samples were incubated at room temperature for 15 min, and SOD activity was calculated as: SOD = (Abs standard − Abs sample/Abs standard) × 100 = % of NBT reduction/SOD. Results were expressed in international units (IU) of CuZn-SOD.

### 2.9. Bactericidal Assay

Enteropathogenic *Escherichia coli* (EPEC, serotype 0111:H2) was used to evaluate phagocytic and microbicidal activity. Bacterial suspensions (10^7^/mL) were incubated with phagocytes at 37 °C for 30 min. Extracellular bacteria were removed by centrifugation, and cells were stained with acridine orange (14.4 g/L, Sigma-Aldrich, St. Louis, MO, USA) for 1 min. Phagocytosis was analyzed by counting 100 cells under immunofluorescence microscopy (400× and 1000×). The phagocytosis index was calculated by counting the number of cells ingesting at least 3 bacteria in a pool of 100 cells. To determine the bactericidal index, we stained the slides with acridine orange and counted 100 cells with phagocytized bacteria. The bactericidal index is calculated as the ratio of orange-stained (dead) to green-stained (alive) bacteria × 100 [19]. All the experiments were performed in duplicate.

### 2.10. Intracellular Ca^2+^ Release Determination

We performed fluorescence staining using a FACS Calibur flow cytometer (BD, San Jose, CA, USA) to assess intracellular Ca^2+^ release in MN cells [20]. Cells were loaded with the fluorescent calcium indicator Fluo-3 acetoxymethyl ester (Fluo-3 AM; Sigma, St. Louis, MO, USA). Cell suspensions, either pretreated or not with 50 μL of resistin (Sigma, St. Louis, MO, USA; final concentration, 100 ng/mL) were incubated with 5 μL of Fluo-3 (1 μg/mL) for 30 min at 37 °C, followed by two additional washes in PBS/BSA (5 mg/mL; 160× *g*, 10 min, 4 °C). Samples were then analyzed by flow cytometry (FACS Calibur system, BD, San Jose, CA, USA). The intracellular Ca^2+^ release was expressed as the geometric mean fluorescence intensity (MFI) of Fluo-3.

### 2.11. Statistical Analysis

Statistical analyses were performed using BioEstat^®^ version 5.0 software (Mamirauá Institute, Belém, Brazil). Data are presented as mean ± standard deviation. The D’Agostino test was used to assess data normality, and the Levene test was applied to verify the homogeneity of variances before performing the analysis of variance. The sample size was estimated assuming a statistical power of 80% (β = 0.20) and a significance level of 5% (α = 0.05). Differences in resistin levels, superoxide release, SOD activity, phagocytosis, bactericidal activity, and intracellular Ca^2+^ release were analyzed using one-way analysis of variance (ANOVA) followed by the Bonferroni Post Hoc test for group comparisons. Statistical significance was set at *p* < 0.05.

## 3. Results

Table 1 summarizes the clinical characteristics of mothers according to weight status. Maternal age and gestational age were similar among groups, although obese mothers tended to be slightly older (31.7 ± 4.8 years) and to deliver earlier (37.2 ± 3.5 weeks) than eutrophic women (29.5 ± 5.1 years; 39.8 ± 2.6 weeks).

As expected, BMI values differed significantly between groups in both the first and third trimesters (*p* < 0.001). Eutrophic mothers had a mean BMI of 23.7 ± 2.1 kg/m^2^ in the first trimester and 33.6 ± 4.2 kg/m^2^ in the third trimester, whereas obese mothers reached 34.7 ± 5.1 kg/m^2^ and 41.1 ± 4.5 kg/m^2^, respectively.

Fasting glucose levels were slightly higher in overweight and obese women (92 ± 5.7 mg/dL and 94.7 ± 7.5 mg/dL, respectively) compared with eutrophic mothers (85.5 ± 4.6 mg/dL), though all remained within the normal range. Hypertension was observed only among overweight (18%) and obese (26%) mothers, while no cases of diabetes were reported in any group.

A similar proportion of mothers in all groups reported regular physical exercise (39% in eutrophic, 33% in overweight, 41% in obese), indicating comparable levels of physical activity during pregnancy.

### 3.1. Resistin Levels

**Figure 1:**
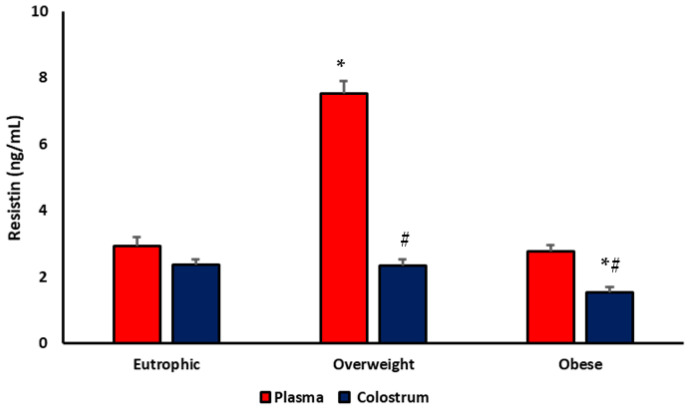
Concentration of resistin (ng/mL) in plasma and colostrum supernatant from eutrophic, overweight, and obese mothers. * *p* < 0.05 indicates differences among groups (eutrophic, overweight, and obese) for the same sample type (plasma or colostrum); # *p* < 0.05 indicates differences between plasma and colostrum within the same group.

Resistin concentrations varied according to the maternal weight status and the biological sample analyzed. As shown in Figure 1, plasma resistin levels were significantly higher in overweight mothers compared with eutrophic and obese groups (*p* < 0.05). In contrast, colostrum resistin levels were significantly lower in obese mothers compared with eutrophic and overweight mothers (*p* < 0.05). When comparing the two sample types within each group, resistin concentrations were consistently lower in colostrum supernatant than in plasma in overweight and obese mothers (#*p* < 0.05).

### 3.2. Superoxide Release

**Figure 2:**
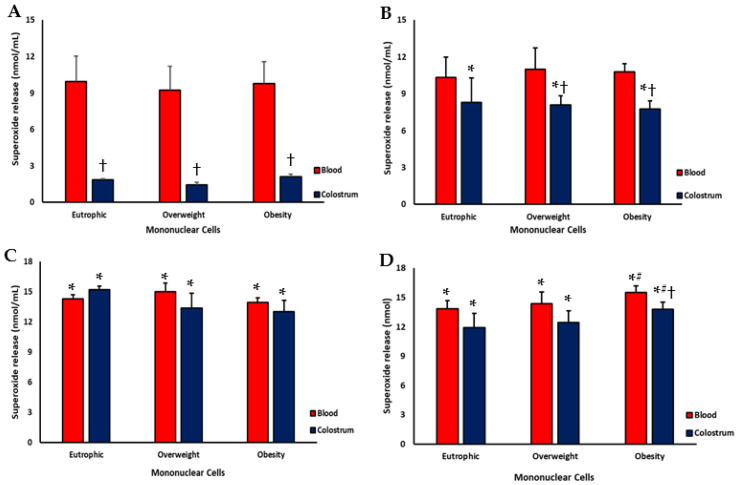
Superoxide release by blood and colostrum cells from eutrophic, overweight, and obese mothers treated or not with resistin in the presence of *Enteropathogenic Escherichia coli* (EPEC). (**A**) PBS (untreated cells-control); (**B**) Cells-EPEC; (**C**) Cells-Resistin; (**D**) Cells-Resistin plus EPEC. Data are expressed as mean ± standard error of the mean (SEM). Statistical analysis was performed using one-way ANOVA (*p* < 0.05). * indicates comparison between untreated cells (PBS) and cells incubated with resistin e/or EPEC, within the same group; # indicates comparison between maternal groups for the same treatment and sample type; † indicates comparison between sample types (blood vs. colostrum) within the same treatment and group.

Spontaneous superoxide release was significantly lower in colostrum phagocytes than in maternal blood phagocytes, regardless of maternal weight status (Figure 2A). After bacterial stimulation, colostrum phagocytes exhibited a marked increase in superoxide production compared with their spontaneous levels. When comparing groups, colostrum phagocytes from overweight and obese mothers released lower levels of superoxide than maternal blood cells from the same group. Figure 2B).

Treatment with resistin, regardless of maternal weight status, significantly increased superoxide release in phagocytes from both colostrum and blood compared with untreated cells (Figure 2C). Following the EPEC challenge, resistin-treated phagocytes exhibited a further enhancement in superoxide production. The highest superoxide levels were detected in blood phagocytes treated with resistin and subsequently stimulated with EPEC (Figure 2D).

### 3.3. Superoxide Dismutase (SOD)

**Figure 3:**
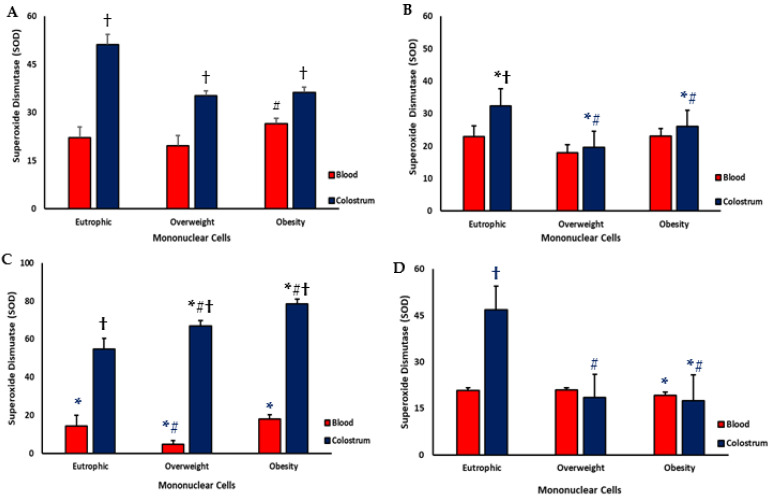
Superoxide dismutase (SOD) concentrations in the culture supernatant of blood and colostrum cells from eutrophic, overweight, and obese mothers treated or not with resistin in the presence of *Enteropathogenic Escherichia coli* (EPEC. (**A**) PBS (untreated cells-control); (**B**) Cells-EPEC; (**C**) Cells-Resistin; (**D**) Cells-Resistin plus EPEC. Data are expressed as mean ± standard error of the mean (SEM). Statistical analysis was performed using one-way ANOVA (*p* < 0.05). * indicates comparison between untreated cells (PBS) and cells incubated with resistin e/or EPEC, within the same group; # indicates comparison between weight-status groups for the same treatment and sample type; † indicates comparison between sample types (blood vs. colostrum) within the same treatment and group.

Figure 3 shows superoxide dismutase (SOD) concentrations in the culture supernatant from maternal blood and colostrum cells, by weight status and treatment condition. Basal (untreated) SOD levels were significantly higher in colostrum than in blood across all groups. Among blood samples, obese mothers exhibited higher basal SOD activity compared with eutrophic and overweight mothers (Figure 3A).

Following bacterial stimulation (EPEC), SOD concentrations significantly decreased in colostrum supernatants from all groups, with the lowest levels observed in cultures from overweight and obese mothers (Figure 3B). In eutrophic mothers, however, supernatant of colostrum-derived cells maintained higher SOD levels than blood cells (Figure 3B).

Treatment with resistin led to a marked reduction in SOD concentration in the supernatant of blood cell cultures compared with basal levels, with the lowest values observed in overweight mothers (Figure 3C). In contrast, in colostrum cell cultures, resistin increased SOD levels in both overweight and obese groups. In all experimental conditions, SOD concentrations remained higher in colostrum than in blood cultures (Figure 3C).

When resistin was combined with EPEC, SOD concentrations decreased in both blood and colostrum cultures from obese mothers (Figure 3D). In colostrum, SOD activity was significantly lower in the overweight and obese groups compared with eutrophic mothers. In contrast, the highest SOD levels were observed in colostrum from eutrophic mothers treated with resistin plus EPEC.

#### Phagocytosis and Microbicidal Activities of Blood and Colostrum Phagocytes

**Figure 4:**
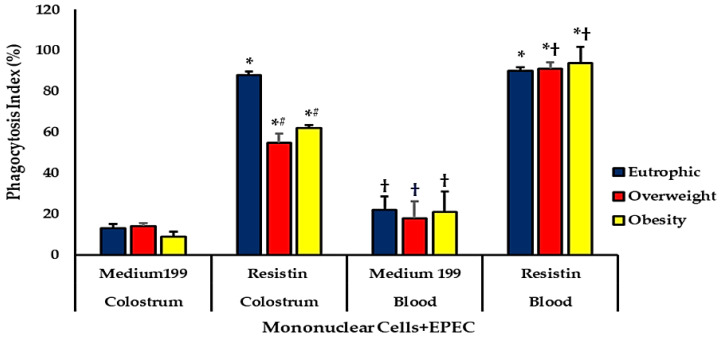
Phagocytosis index (%) of colostrum and blood mononuclear (MN) cells treated or not with resistin, from eutrophic, overweight, and obese mothers. Data are expressed as mean ± standard error (ANOVA, *p* < 0.05). * Comparison between untreated and resistin-treated cells within the same group and sample type; # comparison among groups under the same treatment and sample type; † comparison between sample types under the same treatment and group.

Phagocytosis of mononuclear (MN) cells treated with resistin, derived from both colostrum and blood, was increased in all groups compared with untreated cells. Among untreated samples, blood cells exhibited greater phagocytic activity than those from colostrum. In colostrum, resistin treatment reduced phagocytosis rates in overweight and obese mothers compared with eutrophic mothers. Conversely, blood cells from overweight and obese mothers treated with resistin displayed higher phagocytic activity than their respective colostrum cells (Figure 4).

In blood samples, resistin treatment increased the microbicidal index of cells from overweight and obese mothers. In contrast, in colostrum, cells from eutrophic mothers treated with resistin exhibited higher microbicidal activity. When comparing sample types, resistin-treated colostrum cells from eutrophic mothers showed the highest microbicidal indexes.

**Figure 5:**
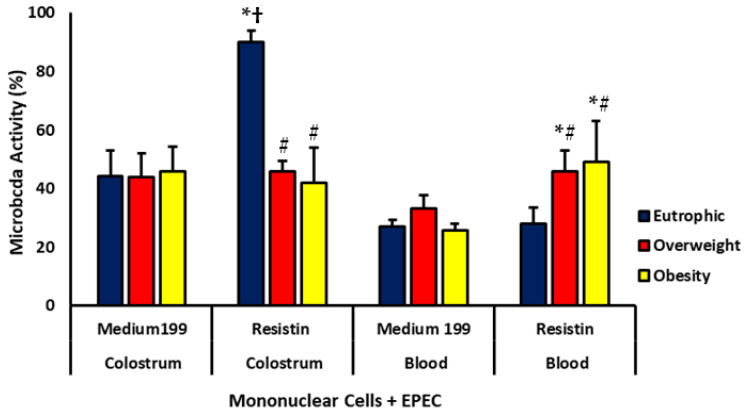
Microbicidal index of colostrum and blood mononuclear (MN) cells treated or not with resistin and exposed to *E. coli* (EPEC) from eutrophic, overweight, and obese mothers. Data are expressed as mean ± standard error (ANOVA, *p* < 0.05). * Comparison between untreated and resistin-treated cells within the same group and sample type; # comparison among groups under the same treatment and sample type; † comparison between sample types under the same treatment and group.

Within-group analysis revealed that, in colostrum, resistin-treated cells from overweight and obese mothers showed lower microbicidal activity than those from eutrophic mothers. Conversely, in blood samples, mononuclear cells treated with resistin showed higher microbicidal indexes in the overweight and obese groups (Figure 5).

**Figure 6:**
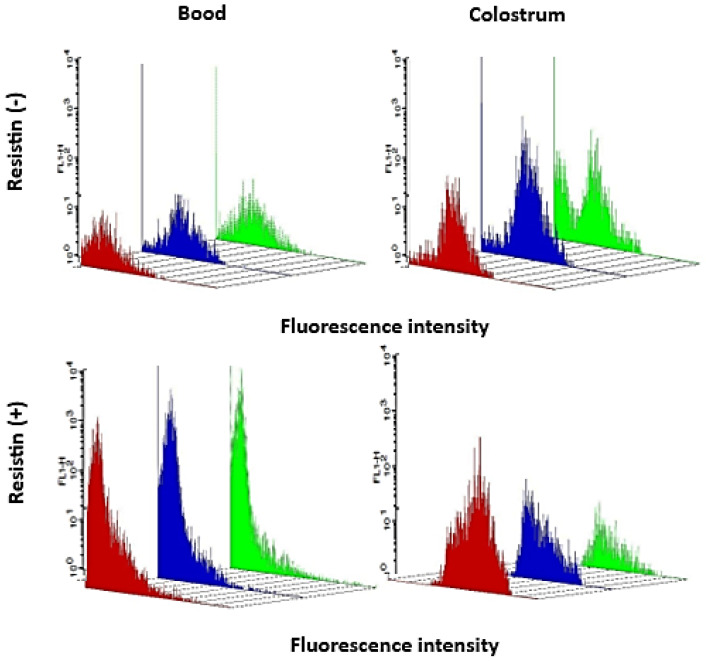
Three-dimensional image of fluorescence intensity (FL-1) of intracellular Ca^2+^ release in the colostrum and blood MN cells treated or not with resistin from mothers with overweight or obesity. Flow Cytometry (FACScalibur, Becton, Dickinson, Franklin Lakes, NJ, USA) was used to evaluate microsphere size and immunofluorescence—red color: eutrophic; blue color: overweight; green color: obese.

Table 2 presents the intracellular Ca^2+^ release in the presence of resistin, while Figure 6 displays the three-dimensional fluorescence intensity of intracellular Ca^2+^ release in the studied groups. In colostrum cells, the adipokine increased intracellular Ca^2+^ release in the eutrophic group but decreased it in the obese group. Resistin also enhanced intracellular Ca^2+^ release in blood phagocytes from overweight mothers. Among untreated cells, intracellular Ca^2+^ release was higher in colostrum than in blood phagocytes within the overweight group. Conversely, both colostrum and blood phagocytes from obese mothers exhibited reduced intracellular Ca^2+^ release in response to resistin.

**Figure 7:**
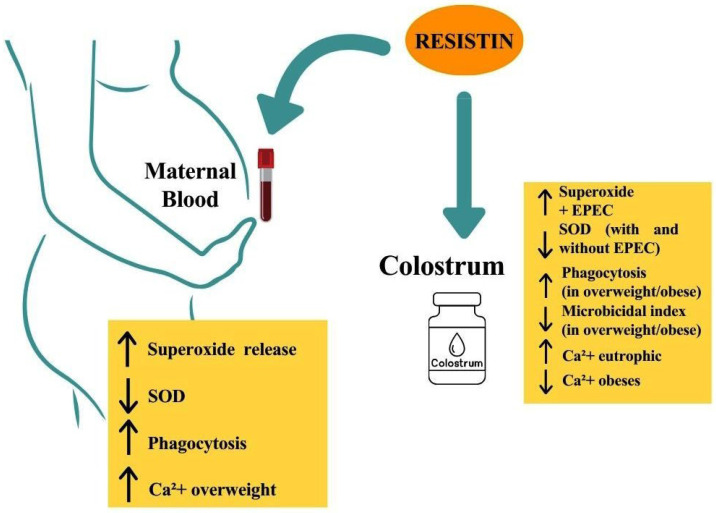
The schematic representation summarizes the main findings of the effects of resistin on the functional activity of mononuclear cells in colostrum and blood from mothers with overweight and obesity.

The schematic representation summarizing the main findings in colostrum and blood from mothers is illustrated in Figure 7.

## 4. Discussion

Resistin, a low-molecular-weight adipokine (~12.5 kDa), plays an important role in the crosstalk between metabolism and inflammation. Adipocytes and immune cells secrete it, acting as a proinflammatory mediator that links metabolic dysfunction to innate immune activation [21,22]. In this study, we found reduced resistin concentrations in the colostrum of obese mothers and higher plasma levels in overweight women, suggesting compartment-specific regulation of resistin secretion during lactation. These findings are consistent with previous evidence showing elevated circulating resistin in overweight and obese individuals, associated with chronic low-grade inflammation, insulin resistance, and early metabolic imbalance [23,24].

Breast tissue dysfunction and altered cytokine signaling may explain these differences. Obesity has been shown to impair the synthesis and secretion of adipokines such as resistin and adiponectin in breast milk [1,25,26,27], both of which participate in feedback mechanisms regulating body weight and energy metabolism. These changes could reflect impaired mammary gland responsiveness to hormonal and inflammatory signals, leading to altered bioactive profiles in colostrum and potentially compromising immunometabolic communication between mother and infant.

The dynamic pattern of resistin secretion throughout lactation supports this interpretation. Previous studies have demonstrated that resistin concentrations are highest in colostrum and decrease over time [27,28], a process influenced by prolactin, estradiol, and inflammatory mediators such as IL-6 and C-reactive protein [29]. In conditions of maternal inflammation or obesity, this regulatory balance can be disrupted. While some reports indicate that resistin levels are independent of BMI, suggesting a direct inflammatory role [29], others show elevated resistin concentrations in severe inflammation [28]. Together, these findings indicate that the mammary resistin response may shift from a regulatory to a maladaptive state depending on the intensity of systemic inflammation.

The lower resistin concentrations observed in the colostrum of obese mothers in this study could therefore result from local oxidative stress and chronic inflammation suppressing resistin synthesis or transport into milk. Structural and functional alterations in mammary epithelial cells associated with obesity can further compromise secretory capacity and modify the colostrum’s bioactive profile [30,31]. These mechanisms are consistent with previous reports describing reduced adipokine transfer and secretory dysfunction in obese women.

Physiologically, resistin in colostrum may contribute to neonatal glucose homeostasis and immune maturation by enhancing hepatic gluconeogenesis and modulating innate immune responses [32]. Therefore, the reduction in colostrum resistin levels among obese mothers may represent a loss of beneficial immunometabolic signaling for the newborn, potentially influencing early metabolic programming. Altogether, these findings reinforce that maternal metabolic and inflammatory status profoundly affects the transfer of bioactive molecules, such as adipokines, cytokines, and growth factors, into breast milk, with potential long-term implications for offspring development [8,33,34].

In addition to these soluble mediators, colostrum provides immune cells that actively contribute to neonatal defense. Macrophages, the main phagocytes in colostrum, play a pivotal role through chemotaxis and phagocytosis [13,19,20]. During this process, they generate reactive oxygen species such as the superoxide anion, which help eliminate pathogens and ensure effective immune protection [20,21,22,23,24,25,26,27,28,29,30,31,32,33,34,35]. Maternal metabolic and inflammatory conditions can influence the functional state of these cells. In this study, superoxide release by colostrum phagocytes was lower, regardless of maternal weight status, indicating that colostrum-derived cells maintain a controlled oxidative profile. After bacterial stimulation, colostrum phagocytes showed increased superoxide production. This regulation can prevent unnecessary damage to mammary gland tissue, preserving antimicrobial activity.

Resistin treatment increased superoxide release in colostrum and blood phagocytes, regardless of maternal weight status. The highest superoxide levels were found in blood phagocytes treated with resistin and subsequently challenged with EPEC, suggesting a synergistic interaction between metabolic and microbial stimuli. These findings reinforce the hypothesis that resistin acts as a modulator of the oxidative burst, possibly by activating and thereby increasing the generation of reactive oxygen species (ROS).

This altered immunometabolic environment can also affect redox homeostasis. Oxygen-derived radicals (ROS) are essential for antimicrobial defense, but excessive ROS can trigger oxidative damage and inflammatory amplification [34,35]. The antioxidant enzyme superoxide dismutase (SOD) serves as a key regulator by converting superoxide radicals into less reactive species, maintaining cellular integrity and limiting oxidative stress [34,35]. Disruptions in this balance may reflect metabolic stress and contribute to impaired immune regulation in the mammary compartment of obese mothers.

In our study, resistin stimulated superoxide generation in both maternal blood and colostrum cells, regardless of weight status, while simultaneously increasing SOD levels in cultures from overweight and obese mothers. This dual effect suggests that resistin may disrupt oxidative–antioxidant equilibrium under metabolic stress conditions [36]. Interestingly, studies have shown that resistin can reduce the toxicity of ROS [37] and SOD activity in vascular cells [38], indicating that its regulatory effect on antioxidant defenses is tissue- and context-dependent. In the present model, the modulation of SOD production by resistin appeared to depend on both the cell type and maternal weight status, possibly reflecting adaptive or compensatory mechanisms to counteract redox imbalance in obese mothers.

Changes in oxidative cellular metabolism can modify intracellular responses, especially phosphorylation events [39], and may be reflected in cellular microbicidal mechanisms [19]. Although resistin increases ROS generation, it can simultaneously compromise antioxidant defenses and modulate phagocytic capacity (especially under bacterial challenge). In humans, resistin plays a key role in inflammatory processes and immune responses. It performs several biological functions, is expressed in various tissues, and participates in metabolic homeostasis [6]. In the present study, blood cell phagocytosis indices, regardless of weight status, increased in the presence of resistin. Still, this increase was not reflected in the microbicidal activity of cells of eutrophic and overweight mothers. Serum resistin levels in normal-weight individuals are associated with systemic inflammation markers [39]. In colostrum, MN cells from obese mothers showed lower phagocytosis rates. When treated with resistin, phagocytosis rates increased in both cell types—colostrum and blood—regardless of maternal weight status. However, resistin-treated colostrum MN cells from overweight and obese mothers showed lower phagocytic rates than those from normal-weight mothers.

These findings suggest that resistin stimulates phagocytic activity, consistent with its role as a proinflammatory adipokine that modulates immune cell responses [40,41]. However, the reduced phagocytic activity observed in colostrum cells from overweight and obese mothers may reflect an altered functional state of these cells, possibly related to the metabolic and inflammatory dysregulation associated with maternal obesity. Previous studies have shown that obesity can impair macrophage activation and reduce the efficiency of pathogen recognition and phagocytosis [42,43].

These data indicate that, although resistin can increase the phagocytic potential of mononuclear cells, the magnitude of this response depends on maternal metabolic status. Chronic low-grade inflammation, characteristic of obesity, may attenuate the beneficial immunostimulatory effects of resistin, leading to a less effective immune response in the colostrum environment.

The effect of resistin on the phagocytic function of maternal mononuclear cells modulated the microbicidal activity of both blood and colostrum cells. Resistin increased the microbicidal activity of colostrum cells from normal-weight and obese mothers, whereas it reduced this activity in cells from overweight mothers. Previous studies have shown that adiponectin, leptin [33], and melatonin [40] can enhance the microbicidal activity of colostrum phagocytes from overweight mothers, improving their antimicrobial capacity. In contrast, in our study, resistin exerted a more pronounced stimulatory effect on cells from normal-weight and obese mothers.

Alterations in resistin-induced microbicidal activity and apoptosis have previously been reported in colostrum macrophages from mothers with diabetes-associated obesity [8], as well as changes in cytokine profiles and metabolites in colostrum from obese mothers [9]. These findings support the hypothesis that maternal metabolic stress impairs colostrum immune function, indicating that disturbances in the maternal metabolic environment directly affect its immunometabolic markers.

In the present study, resistin treatment increased the microbicidal index in blood mononuclear cells from overweight and obese mothers, while eutrophic mothers exhibited a greater microbicidal response in colostrum cells. This pattern suggests that resistin may exert tissue-specific and condition-dependent effects on innate immune cells, modulating their antimicrobial capacity according to the metabolic and inflammatory milieu.

In obesity, chronic low-grade inflammation and elevated basal expression of Toll-like receptors and NF-κB signaling components may prime monocytes and macrophages for a stronger proinflammatory and microbicidal response to resistin stimulation [44,45]. Resistin has been described as an amplifier of macrophage activation, enhancing reactive oxygen species generation and phagocytic capacity via ERK and p38 MAPK pathways [36,37]. Furthermore, previous studies demonstrated that resistin increases microbicidal and phagocytic activity in macrophages from the colostrum of diabetic mothers, suggesting a direct immunomodulatory effect dependent on the maternal metabolic environment [8]. Resistin also modulates cytokine release, particularly IL-6 and TNF-α, reinforcing its dual role as a metabolic and inflammatory signal [8].

Thus, the greater microbicidal activity observed in the blood cells of overweight and obese mothers may reflect enhanced sensitivity or a preactivated immune state in response to resistin. Conversely, in the mammary compartment, the metabolic and hormonal environment may limit responsiveness to resistin, leading to lower microbicidal activity in colostrum cells from obese mothers compared with eutrophic mothers [38,39]. Taken together, these results indicate that resistin differentially modulates innate immune responses depending on both the maternal metabolic state and the tissue microenvironment.

The microbicidal activity and the products derived from oxidative metabolism promoted by resistin can have important clinical implications. Increased superoxide alters intracellular Ca^2+^ signaling and phosphorylation events during oxidative metabolism [46,47]. The study’s findings indicate that resistin modulates intracellular Ca^2+^ release in maternal phagocytes, with this effect dependent on weight status. In colostrum cells from eutrophic mothers, resistin increased Ca^2+^ mobilization, suggesting preserved signaling pathways that enhance cell activation and innate immune responses. Conversely, the reduced Ca^2+^ release in phagocytes from obese mothers suggests impaired intracellular signaling, possibly due to chronic exposure to adipokines. These data suggest that maternal adiposity alters phagocyte responsiveness to resistin, impairing Ca^2^-dependent activation and potentially altering the immunoprotective capacity of colostrum. Further studies should explore how restoring metabolic homeostasis might reestablish proper Ca^2+^ signaling and immune function during lactation.

Resistin also increased Ca^2+^ release in blood phagocytes from overweight mothers, suggesting an intermediate state of immune hyperresponsiveness. Studies report that endoplasmic reticulum stress may regulate phagosomal Ca^2+^ flux and macrophage microbicidal activity [48]. Furthermore, the higher basal Ca^2+^ level in untreated colostrum phagocytes reinforces their naturally activated profile, shaped by the local cytokine and hormonal environment during lactation [49]. Resistin has been described as a key mediator of the crosstalk between metabolism and immunity [50,51]. In obesity, however, this signaling balance is disrupted, leading to reduced Ca^2+^ responsiveness and immune dysfunction.

It should also be noted that there is a positive relationship between maternal and umbilical cord resistin levels, suggesting that the placenta may secrete resistin into the fetal circulation, as it does in the maternal circulation [26]. Future studies are needed to understand better the mechanisms by which this adipokine modulates mother-child interactions during pregnancy and lactation. Collectively, these results highlight resistin as a key adipokine at the interface between maternal metabolism and neonatal immune programming.

## 5. Conclusions

In conclusion, our findings indicate that resistin modulates cellular oxidative metabolism as well as the phagocytic and microbicidal activities and intracellular Ca^2+^ release of both blood and colostrum phagocytes, regardless of maternal weight status. Notably, the elevated plasma resistin levels observed in overweight mothers may represent an adaptive mechanism during gestation, potentially influencing the metabolic programming of their offspring and contributing to improved body weight regulation later in life.

## Figures and Tables

**Table 1:** Clinical data of mothers with different glycemic and weight statuses.

Parameters	Eutrophic (*n* = 28)	Overweight (*n* = 27)	Obese (*n*= 27)
Age (years)	29.5 ± 5.1	28.6 ± 4.5	31.7 ± 4.8
Gestational age (weeks)	39.8 ± 2.6	37.8 ± 3.5	37.2 ± 3.5
BMI-1	23.7 ± 2.1	27.2 ± 3.1	34.7 ± 5.1
BMI-2	33.6 ± 4.2	38.2 ± 4.8	41.1 ± 4.5
Fasting glucose	85.5 ± 4.6	92 ± 5.7	94.7 ± 7.5
Hypertension (%)	0	5 (18%)	7 (26%)
Diabetes (%)	0	0	0
Physical exercise (%)	11 (39%)	9 (33%)	11 (41%)

Notes: Values are presented as mean ± standard deviation (SD) or as percentages, as appropriate. BMI-1 = body mass index measured in the first trimester of pregnancy (kg/m^2^); BMI-2 = body mass index measured in the third trimester of pregnancy (kg/m^2^). Hypertension refers to clinically diagnosed gestational or chronic hypertension. Physical exercise refers to self-reported regular moderate activity (≥3 times per week).

**Table 2:** Intracellular Ca^2+^ release by colostrum and blood mononuclear [MN] cells is indicated by fluorescence intensity.

MN Cells Incubated	Sample	Eutrophic	Overweight	Obesity
Fluorescence Intensity [%]				
PBS	Blood	27.0 ± 2.2	23.3 ± 2.1	25.8 ± 2.5
	Colostrum	25.9 ± 1.8	29.5 ± 3.0 †	29.9 ± 2.8
Resistin	Blood	30.2 ± 3.5	29.3 ± 3.5 *	20.8 ± 2.5 #
	Colostrum	34.2 ± 2.9 *	25.4 ± 2.4	16.4 ± 3.6 *#

Notes: The Data are expressed as mean ± standard error (ANOVA, *p* < 0.05). * Comparison between untreated and resistin-treated cells within the same group and sample type; # comparison among groups under the same treatment and sample type; † comparison between sample types under the same treatment and group.

## Data Availability

The data supporting the findings of this study are available from the corresponding authors upon reasonable request.
